# Childhood Mortality by Parental Cause of Death

**DOI:** 10.1001/jamanetworkopen.2026.2790

**Published:** 2026-03-23

**Authors:** Sean Esteban McCabe, Luisa Kcomt, Rebecca J. Evans-Polce, Glenn Radford, Samuel D. Tennant, Eric Hulsey, Vita V. McCabe

**Affiliations:** 1 Center for the Study of Drugs, Alcohol, Smoking and Health, University of Michigan, Ann Arbor; 2 Survey Research Center, Institute for Social Research, University of Michigan, Ann Arbor; 3 Institute for Healthcare Policy and Innovation, University of Michigan, Ann Arbor; 4 School of Social Work, Wayne State University, Detroit, Michigan; 5 Division for Vital Records and Health Statistics, Michigan Department of Health and Human Services, Lansing; 6 School of Public Health, University of Michigan, Ann Arbor; 7 Institute for Research, Education and Training in Addictions, Pittsburgh, Pennsylvania; 8 Addiction Center, Department of Psychiatry, University of Michigan, Ann Arbor

## Abstract

This cohort study examines rates of childhood mortality in offspring by cause of parental death (ie, drug overdose, homicide, or suicide) to evaluate the need for preventive interventions for bereaved children.

## Introduction

US parental mortality has reached historic high levels in recent years, increasingly associated with 3 leading preventable causes of death: drug overdose, homicide, and suicide.^1,2^ Parental mortality has a major impact on the emotional, social, and mental well-being of bereaved children and can reduce their level of protection against harm, thus increasing the risk of early mortality.^3,4,5^ Building on previous parental mortality research,^3,4,5^ this study examines rates of childhood mortality in bereaved offspring by cause of parental death (ie, drug overdose, homicide, or suicide) to help evaluate the need for preventive interventions for bereaved children.

## Methods

This cohort study was determined to be exempt from review and the need for informed consent by the University of Michigan’s institutional review board, in accordance with 45 CFR §46. This report follows the STROBE reporting guidelines for cohort studies. In collaboration with the Division for Vital Records and Health Statistics at the Michigan Department of Health and Human Services, death certificate records were used to identify people who died statewide in Michigan between January 2009 and December 2023. Individuals who were also listed on birth certificate records as a biological mother or father were used to identify children born during 1992 to 2023 who appeared on these birth certificates and were collated by year of parental death, year of the child’s birth, and parental death cause. *International Statistical Classification of Diseases and Related Health Problems, Tenth Revision* codes were used to determine cause of parental death—homicide, suicide, and drug overdose or drug related—consistent with prior methods.^1,4^ This established a dynamic cohort of children aged 17 years or younger who experienced the death of a biological parent due to homicide, suicide, and drug overdose or drug-related causes during childhood. Death certificate records were used from this cohort to identify childhood mortality cases as a function of cause of parental death. Group-specific mortality rates of parentally bereaved children from each cause of death were calculated by dividing the number of bereaved children who died by parental deaths between 2009 and 2023, multiplied by 10 000. Score tests compared observed group-specific mortality counts to an expected group count under the overall 2009 to 2023 Michigan child mortality rate of 5.22 deaths per 10 000 (α = .05).^6^ Excess deaths were calculated as the difference between the observed group mortality count and the count expected under that overall rate. Data were analyzed using R statistical software version 4.5.1 (R Project for Statistical Computing). Statistical significance was set at 2-sided *P* < .05 using a *z* test.

## Results

Between 2009 and 2023, 32 262 children aged 17 years or younger in Michigan experienced parental deaths due to homicide, suicide, or drug overdose or drug-related causes (Table). Childhood mortality rates were significantly higher among bereaved children who experienced parental deaths from homicide, suicide and drug overdose or drug-related causes compared with all Michigan children. Bereaved children who experienced parental death due to homicide had the highest rate of childhood mortality (106.10 deaths per 10 000) followed by suicide (66.16 deaths per 10 000) and drug overdose or drug-related causes (36.97 deaths per 10 000), accounting for nearly 150 excess child deaths (Table).

**Table.** Mortality Rates Among Bereaved Children by Cause of Parental Death^a^

Cause of parental death	No. of parental deaths	No. of child deaths in parentally bereaved children	Bereaved child death/parental death, rate per 10 000^b^	No. of excess child deaths
Drug overdose or drug-related	21 368	79	36.97	67.85
Suicide	6651	44	66.16	40.53
Homicide	4243	45	106.10	42.79

^a^ Child death is defined as death occurring at age 17 years or younger. Between 2009 and 2023, the overall crude child death rate for all of Michigan children aged 17 years or younger was 5.22 child deaths per 10 000 population. Data are from the Division for Vital Records & Health Statistics, Michigan Department of Health & Human Services, and the Monthly Vital Statistics Reports, National Center for Health Statistics.

^b^ Data in this column are group-specific childhood mortality rates for each cause of parental death that are significantly different from the expected 2009 to 2023 Michigan child mortality rate of 5.22 per 10 000 (*P* < .001).^6^

## Discussion

To our knowledge, this statewide cohort study is the first of its kind and reveals that childhood mortality is significantly higher among children bereaved by parental drug overdose, homicide, and suicide compared with the general child population. Parental homicide was associated with the highest risk of mortality in children and highlights the need for research into potential explanations, such as the impact on family restructuring and mental health.^2,3,4,5^ Although it provides critical insights, the study has limitations, including provisional 2023 data, a focus solely on biological parents, and potential underreporting of paternal deaths, suggesting the actual impact is likely higher. The study does not establish causation and calls for future research using causal inference methods to examine confounders like family restructuring and identification of bereaved children’s causes of death. The findings indicate that preventing parental deaths from overdoses, suicides, and homicides would be associated with lower mortality among children. Researchers are encouraged to replicate this study to pinpoint at-risk bereaved children, identify bereavement service deserts, and address racial and ethnic differences.^2^ Early preventive interventions and childhood bereavement services are critical for the growing population of children experiencing parental deaths.

## Notes

Supplement. Data Sharing Statement
