# Supplementary material for: Childhood Mortality by Parental Cause of Death
Source: JAMA Netw Open. 2026 Mar 23;9(3):e262790. doi: 10.1001/jamanetworkopen.2026.2790 (PMC13010188; doi:10.1001/jamanetworkopen.2026.2790)
Supplement: Supplement. — Data Sharing Statement [file jamanetwopen-e262790-s001.pdf]

Data Sharing Statement

McCabe. Childhood Mortality by Parental Cause of Death. *JAMA Netw Open*. Published March 23, 2026. doi:10.1001/jamanetworkopen.2026.2790

Data
Data available: No

Additional Information
Explanation for why data not available: The data is restricted and cannot be released directly to the public. A data use agreement is required to gain access.
